# The use of the *Decision Regret Scale* in non-clinical contexts

**DOI:** 10.3389/fpsyg.2022.945669

**Published:** 2022-09-15

**Authors:** Pierluigi Diotaiuti, Giuseppe Valente, Stefania Mancone, Angela Grambone, Andrea Chirico, Fabio Lucidi

**Affiliations:** ^1^Department of Human Sciences, Society and Health, University of Cassino, Cassino, Italy; ^2^Department of Psychology of Development and Socialization Processes, Sapienza University of Rome, Rome, Italy

**Keywords:** decision-making, *Decision Regret Scale*, regret, validation, gender invariance

## Abstract

The Decision Regret Scale (DRS) was assessed for its psychometric qualities in measuring decision regret in ordinary life scenarios. Although the scale has typically been used with patients and in the context of medical decision-making in earlier studies, this contribution shows that the instrument may have a variety of uses, retaining excellent metric properties even in non-medical contexts. The tool showed good fits with both the CFA and the gender Measurement Invariance. A non-probabilistic selection of 2,534 Italian university students was conducted. The internal consistency measures were found to be completely appropriate. Correlations with the *General Decision-Making Style* (GDMS) and *Scale of Regulatory Modes* were used to check for convergent validity (SRM). Convergence analysis showed that participants with higher regret scores were those who favored a rational decision-making style, while lower regret scores correlated with avoidant and spontaneous styles. With regard to the regulatory modes, the relationship between regret and locomotion was positive. Overall, the directions of association point to an interesting predictive measure of a person’s decision-making and self-regulatory orientation through the evaluation of regret using the DRS. The excellent psychometric properties found foreshadow a reliable use in various contexts where knowledge of post-decisional attitude becomes important: school, university, professional orientation, marketing studies, relationship choices, as well as for use in research.

## Introduction

Regret is the negative state of mind we experience when we realize that if we had made a different decision, we would have gotten a better result (Coricelli et al., 2005; Mazzocco, 2008). The severity of the regret feeling changes with the availability of counterfactual alternatives (Seta et al., 2008). Indeed, research has revealed that there is a propensity to have emotional reactions to situations for which an alternative result is simpler to envision, a process known as emotional amplification (Kahneman and Miller, 1986). The most essential criteria for the development of regret, according to research, are the proximity of the alternative result, the action-non-action component, and the impression of responsibility (Zeelenberg et al., 1998; Byrne and McEleney, 2000; Papé and Martinez, 2017). According to Miller and Taylor (1995), emotions such as regret arise not only when the negative outcome is due to inappropriate or irrational decisions or actions, but whenever there is a counterfactual alternative to one’s actions that is highly available, such as in situations in which one came very close to obtaining a better outcome. Gilovich and Medvec (1994) observed that people more frequently recall regrets associated with actions in the short term (e.g., last week), but in the long term (e.g., last year) situations of regret associated with non-actions are cited more frequently. Sugden (1985) attributes responsibility as a key role for regret. According to his definition, regret consists of two components, the first concerns the evaluation of one’s choice on the basis of the comparison between the actual outcome and the counterfactual outcomes, and the second is given by feelings of responsibility and self-blame and the subjective evaluation of the quality of one’s choice. According to Sugden, the intensity of regret would depend precisely on how responsible we hold ourselves for the choice we make. The impacts of regret on decision-making have also been the topic of significant research aimed at the function of regret anticipation; the idea that individuals can anticipate the remorse they expect to experience as a result of their decisions and use this knowledge as a compass for decision-making has been the focus of several experimental examinations (e.g., Zeelenberg and Pieters, 1999; Hetts et al., 2000; McConnell et al., 2000; Hoelzl and Loewenstein, 2005; Wong and Kwong, 2007).

Over time, several instruments for the measurement of regret have been validated: the *Anticipated Regret Questionnaire* (Godin et al., 2005); the *Anticipated Regret Scale* (Sheeran and Orbell, 1999); the *Decision Regret Scale* (DRS) (Brehaut et al., 2003); the *Experienced Regret Scale* (Keaveney et al., 2007); the *Regret and Disappointment Scale* (Marcatto and Ferrante, 2008); the *Regret Experience Measure* (Creyer and Ross, 1999); the *Regret and Maximization Scale* (Schwartz et al., 2002); the *Regret Measurement* (Tsiros, 1998). The instruments were developed in Australia (Creyer and Ross, 1999), Canada (Brehaut et al., 2003; Godin et al., 2005), Italy (Marcatto and Ferrante, 2008), the United Kingdom (Sheeran and Orbell, 1999) and the United States (Tsiros, 1998; Schwartz et al., 2002; Keaveney et al., 2007). Among these instruments, Brehaut et al. (2003) DRS has some interesting and robust psychometric features (such as being a brief single-factor model with high internal consistency and stability over time) reaffirmed in the studies that have employed it, both in its original form and in the form validated for other national contexts (Bonaccio and Girard, 2015; Becerra Pérez et al., 2016; Tanno et al., 2016; Advani et al., 2019; Calderon et al., 2019; Haun et al., 2019; Wilding et al., 2020; Xu et al., 2020; Telatar et al., 2021; Liu et al., 2022). So far, the use of the tool has been aimed at assessing regret in patients who have already made a medical decision. Brehaut et al. (2003) reported the results of four separate patient groups: postmenopausal women contemplating hormone replacement therapy, males considering prostate cancer treatment, women considering breast cancer treatment, and women considering breast cancer adjuvant treatment. DRS was also utilized with breast cancer patients in another trial (Goel et al., 2001).

The DRS has been translated into seven languages (so far, with the exception of Italian) and customized for use in a variety of cultural settings (Becerra Pérez et al., 2016). This same single-factor structure was confirmed in the Chinese validation (Xu et al., 2020), as posited in the original English version, and this was also mentioned in previous studies, such as the one in the United States that assessed DRS accuracy in patients undergoing a subcutaneous defibrillator, and another that investigated the validity of the DRS Japanese version (Tanno et al., 2016; Calderon et al., 2019).

Although earlier research have mostly employed the DRS scale with patients when it comes to medical decisions, this our contribution intends to show that the instrument may be used also in a variety of contexts while maintaining its strong metric features. On the Italian psychometric scene, further instruments for assessing general regret would be useful, as only the Marcatto and Ferrante (2008) scale is now available, with significant limits in its application mentioned in the literature. In fact, as already reported by Buchanan et al. (2016), the scale includes distinct items focused on affect and on counterfactuals, but it contains only two items specific to regret alone, both of which focus on the cognitive component. Therefore, the main objective of our study was to propose a first validation in the Italian context of the use of the DRS to measure post-decisional regret starting from choice situations proper to everyday life. Evaluation of the maintenance of the psychometric qualities of the instrument in non-medical settings was done through the use of both exploratory (EFA) and confirmatory (CFA) analyses, which was followed by a measure of scale convergence with constructs of decision-making and regulatory orientation of the person. The contributions of Ordóñez and Connolly (2000) and Zeelenberg et al. (2000), and more recently Gaafar et al. (2018) while considering the function of predictors or concurrent variables of regret, indicated a probable link between regret and a person’s decision-making style. Earlier, Ueichi and Kusumi (2004) also presented an observational study in which some relationships between regret and decision-making styles were illustrated. More specifically, they reported that analytic decision-makers tended to cope with their regret by improving their behavior more than intuitive decision-makers. While Jurasova and Spajdel (2011) found that rational decision makers anticipate regret during the decision making process, but they are not good at correctly predicting regret intensity, in comparison with non-rational decision makers. Therefore, taking into account such backgrounds in the literature, in our study, we evaluated the convergence between the DRS and the General Decision-Making Style (Scott and Bruce, 1995) in the Italian version validated by Di Fabio (2007). Our hypothesis was to find a positive association with the rational and intuitive style and a negative association with the avoidant, dependent, and spontaneous style. A second hypothesis was that an association between regret and a person’s regulatory patterns could also be detected., i.e., the way people deal with situations in order to achieve a goal. According to the theory of regulatory modes of Kruglanski et al. (2000), people who are oriented toward the locomotion mode are focused on movement and goal attainment. In contrast, those who are strong in evaluation will compare different goals and analyze different options. In the study by Pierro et al. (2008) it emerged that when faced with a negative outcome of a decision, a high degree of locomotion mode would result in less counterfactual thinking and regret, but a high level of assessment mode would result in the reverse. Panno et al. (2015) showed that people’s regulatory mode affects feelings of regret even before making a decision. In other words, the decision maker’s regulatory mode affects anticipated regret as well as post-decisional regret. People with assessment concerns engage in a greater amount of anticipated regret because they are strategically motivated to make comparisons of all options, when faced with a risky choice, while people with locomotion concerns seem to leave little room triggering anticipated regret because strategically motivated to take the action, when faced with a risky choice (Kruglanski et al., 2000; Lauriola and Levin, 2001). On the basis of this evidence, in our study we also evaluated the convergence of post-decisional regret with the regulatory modes of assessment and locomotion, expecting directions of association similar to those found in Pierro et al. (2008).

## Materials and methods

### Language methods

As per the translation criteria, the DRS version followed forward and backward translations of the original scale (Beaton et al., 2000). Two Italian translators finished the forward translation independently and discussed any discrepancies between the two versions. The resolved Italian version was then provided to two English translators, who back-translated the text separately. Any differences were reviewed and resolved, and changes have been made to the DRS to account for any rewording in order to increase the readability of the items. Ultimately, a focus group of 16 people was formed and arranged to include three distinct age ranges (20–30; 31–40; 41–50), both genders, and individuals with low-medium and high educational degrees. The conversation held on each item following the administration of the tool revealed no comprehensibility or literacy disparities.

### Participants and tools’ administration

The sample size design for the current investigation was predicated on the capacity to validate a sufficient fit of DRS beginning with a translation of the English version, which contained a one-factor model with 5 manifest variables. We were able to identify an effect size of *r* = 0.16 for Pearson product-moment correlation coefficients using a type 1 error of 5% (two tailed), a power of 0.80, and a total sample size of 300 observations. Participants were recruited by forwarding an email contact to students enrolled in a university in central-southern Italy. This email described the study’s objectives as well as its purpose. Subjects were asked to input a specific link located in the same notification, which they then filled out and digitally submitted. Participants were guaranteed of their confidentiality, as well as the use of aggregate data for research purposes. 4,000 contact emails have been sent in total. In terms of the drop-out percentage, 146 participants dropped out after starting to fill it out, resulting in a total of 1,726 returned surveys (851 men and 875 females with an average age of 23.69 and *SD* = 4.08). The protocol involved distributing 16 decision scenario variants among participants, in relation to which they were asked to estimate the regret, if any, experienced in these situations. Convergent validity was assessed with an extra convenient sample of participants obtained online, totaling 808 people (males = 444; females = 364), Mage = 23.54, and *SD* = 4.04. In order to recruit this sample, a total of 2,000 contact emails were sent out. The inclusion criterion was attendance at the University and non-participation in the prior administration. The scenario protocol had the same characteristics as the first one, with the homogeneous distribution of the 16 scenario variants within the 2,000 contact emails. The following Table 1 reports demographic characteristics of the whole sample of participants.

**TABLE 1:** Characteristics of the participants.

Gender	males = 1,295 (49%); females = 1,240 (51%)
Study course	Economy = 305 (12.0%) Foreign languages = 127 (5.0%) Pedagogical sciences = 354 (14.0%) Motor sciences = 177 (7.0%) Law = 405 (16.0%) Humanities = 203 (8.0%) Communication sciences = 152 (6.0%) Engineering = 431 (17.0%) Nursing sciences = 127 (5.0%) Social work = 253 (10.0%)
Year of course	First = 717 (28.3%) Second = 578 (22.8) Third = 514 (20.3%) Fourth = 362 (14.3%) Fifth = 274 (10.8%) Out-of-course = 89 (3.5%)
Father’s education	Primary school = 111 (4.4%) Secondary school = 560 (22.1%) High school diploma = 1,115 (44.0%) University degree = 748 (29.5%)
Mother’s education	Primary school = 215 (8.5%) Secondary school = 598 (23.6%) High school diploma = 1,163 (45.9%) University degree = 558 (22.0%)
Area of residence	City > 50,000 inhabitants = 182 (7.2%) Town < 50,000 inhabitants = 816 (32.2%) Small town < 5,000 inhabitants = 1,536 (60.6%) With family = 1,133 (44.7%) With other students = 1,312 (51.8%) Alone = 89 (3.5%)

### Measures

Decision-making scenario: the participant was asked to identify as closely as possible with an everyday situation involving a choice between two alternatives with different or similar aims, e.g., pursuing a personal utility or fulfilling a previous commitment; renouncing personal pleasure in the name of a higher duty; choosing between two options both pleasant or useful. The person was asked to choose and indicate what he or she was likely to do in that situation. The development of the scenarios was done considering the previous contributions of Jonassen (2012) and Diotaiuti et al. (2020a).

Construction of the scenarios: as the study’s goal was to assess the fit values of the decision regret measure in a non-clinical sample such as university students, it was necessary to propose decision-making situations that students usually encounter, sufficiently diversified as to the nature of the choice. Therefore, four types of alternative choices were initially identified: (1) pursuing a personal pleasure goal (i.e., relaxing while watching a movie) or a personal utility goal (i.e., cleaning the house); (2) pursuing a personal pleasure purpose (i.e., going out for a walk) or a purpose corresponding to an obligation (i.e., paying a tax due); (3) pursuing an aim of personal pleasure (i.e., playing video games) or an aim corresponding to a social expectation (i.e., fulfilling a commitment made to others); (4) pursuing an aim of personal pleasure (i.e., listening to one’s favorite music) or an equally pleasurable aim but with a different content (i.e., going out shopping).

In order to balance a possible positional effect (first alternative, second alternative), three further blocks of scenarios were constructed in which the first alternative was a useful purpose, an obligation, a purpose corresponding to a social expectation, respectively. Thus, as a result of this balancing operation, a total of 16 decision scenario variants were defined.

Among the contact e-mails sent to the students, 250 protocols were sent for each scenario variant, for a total number of 4,000 contact e-mails.

A group of 50 undergraduate students used a preliminary production and classification method to identify 15 action choices that were intended to be pleasant, 18 action choices that were intended to be useful, 12 action choices that were perceived as an obligation, and 13 action choices that were related to commitments made with other people. Following that, students scored each circumstance on a scale of 1–7 to reflect the extent of representativeness of each condition. Eight pleasure circumstances, eight utility conditions, eight obligation conditions, and eight commitment conditions were finally chosen for the procedure based on the better scores achieved.

### Psychometric scales

DRS (Brehaut et al., 2003), consists of “five items answered” on a 5 point bipolar intensity scale and is a unidimensional, self-report measure. Participants rate the item statements by selecting a number between 1 (strongly disagree) and 5 (strongly agree) (strongly disagree). To eliminate acquiescence bias, items 2 and 4 are formulated in a negative manner. After inverting the scores of these two items, the total sum score is calculated by computing the average of the five items, subtracting 1 and multiplying by 25, thus turning the result to a score ranging from 0 to 100. A lower total score suggests fewer regrets, whereas a higher total score indicates more regrets.

*General Decision-Making Style* (GDMS, Scott and Bruce, 1995), in the Italian version by Di Fabio (2007) is made up of 25 items, each having a five-point Likert scale answer (from 1 to 5). It allows to detect five decision-making styles: Rational (item example: “My decisions require careful consideration”); Intuitive (item example: “When I make a decision I trust my instincts”); Dependent (item example: “I rarely make important decisions without consulting other people”); Avoidant (item example: “When I can, I postpone the decision”); Spontaneous (item example: “I make decisions quickly”). Cronbach’s alpha coefficients in relation to the five styles are: 0.73 for Rational; 0.73 for Intuitive; 0.80 for Dependent; 0.84 for Avoidant; 0.78 for Spontaneous. For this investigation, the following criteria were considered to determine reliability: Cronbach’s raw alpha (α) = 0.91 (CIs 95% 0.90; 0.93); McDonald’s omega (ω) = 0.92 (CIs 95% 0.90; 0.93).

*Scale of Regulatory Modes* (SRM) (Higgins et al., 2003; Pierro et al., 2006), consisting of 24 elements (12 for the measure of Assessment Mode and 12 for the measure of Locomotion Mode) 6-point Likert scale (1 = strongly disagree to 6 = strongly agree). Assessment is a comparison component of the Self-regulation system, as it is a propensity to critically examine the current condition in comparison to other options in order to attain our goals in the best possible manner. This study’s reliability measures were the following: α = 0.71; ω = 0.71; (CIs 95% 0.63; 0.77). Locomotion, on the other hand, is a part of our self-adjusting system committed to managing movement by state and maintaining it in order to achieve a goal in a simple and delay-free manner. Reliability measures were the following: α = 0.75; ω = 0.76; (CIs 95% 0.69; 0.81).

## Statistical analysis

In consideration of confirmatory factor analysis (CFA), we adopted recognized conventional criteria and decided that a minimum sample size of 300 persons was necessary (Mundfrom et al., 2005). Assuming a type 1 error of 5% (two-tailed) with a power of 0.80 and a total sample size of 300 observations, we were able to ascertain a Pearson product-moment correlation coefficient effect size of *r* = 0.16.

Verification of the assumptions of univariate and multivariate normality; EFA with Parallel Analysis (PA) as the extraction method; Confirmatory Factorial Analysis (CFA); and evaluation of internal consistency using Cronbach’s alpha coefficient and McDonalds were the primary statistical analyses performed. Composite Reliability Index (CRI) was used to study reliability; values above 0.70 are regarded as satisfactory (Raykov, 1997). Also given were the item-total correlation (> 0.5), the average inter-item correlation (0.15–0.5), and the alpha if an item was eliminated (DeVellis, 2017).

To evaluate the appropriateness of the model, the 10 indices listed below were considered: (1) chi square; (2) the connection between the chi-square value and the degrees of freedom (2/d.f., acceptable values range between 1 and 3); (3) GFI (Goodness of Fit Index), with values greater than 0.90 indicating an acceptable model fit and values greater than 0.95 indicating a good model fit; (4) RMSEA (Root-Mean-Square Error of Approximation), with values between 0.05 and 0.08 indicating an acceptable model fit and values less than 0.05 indicating a good model fit; (5) *p*-value for the test of close fit, with values between 0.50 and 1 indicating an acceptable fit of the model and values between 0.05 and 0.50 indicating a good fit; (6) CFI (Comparative Fit Index) and TLI (Tucker-Lewis Index), with values between 0.95 and 0.97 denoting an acceptable fit of the model and values between 0.95 and 1 indicating a good fit; (7) NFI (Normed Fit Index), with values between 0.90 (Hu and Bentler, 1999; Byrne, 2001; Schermelleh-Engel et al., 2003; Barbaranelli and Ingoglia, 2013).

The factorial structure of the DRS was tested for measurement invariance by gender. As a result, four layered models were evaluated, each with greater degrees of restriction: the base model examined configural invariance and permitted free estimate of all parameters for each group. The metric (weak) invariance model, which was layered within the configural model, adds the constraint of invariant factor loadings between groups to the configural model. The scalar (strong) invariance model, which was layered within the second model, adds the invariant items’ intercept constraint to the comparison groups. Finally, strict invariance was assessed by comparing the scalar model against a model that additionally required residuals to be identical across groups. We concentrated on comparing the CFI, TLI, and RMSEA indices because the Chi-square indices are sensitive to sample size. A variation of these indices more than 0.01 was used as a criteria to rule out the more restrictive model’s invariance and accept the more parsimonious model (Cheung and Rensvold, 2002). The group mean differences in latent variables were examined once the strict invariance was confirmed.

The correlations between the DRS and the components that make up GDMS and SRM were compared to establish convergent validity. Pearson coefficients were used to determine concurrent validity. SPSS version 22 and IBM Amos Graphics 18 were used to conduct statistical analysis.

## Results

Prior to data analysis, the DRS item distribution characteristics of the entire sample were visually examined, and most items had skewness values of 1 or slightly higher. The Mardia Index (average of the squares of the Malhanobis Distances) produced a coefficient (44.86) that was smaller than the limit value (48), proving that the multivariate normality assumptions were correct. Low co-linearity was indicated by low VIF (Variance Inflation Factor) values (<2) and high tolerance values (> 0.60). For residual assumptions verification, the average of the standardized and raw residuals was adjusted to 0; the Durbin–Watson test yielded a value of 1.021, showing the absence of autocorrelation. The metric characteristics of the scale were assessed using an exploratory factor analysis (EFA) and CFA, both of which were designed to examine the quality of the dimensional model of the instrument. To minimize problems of overfitting (Fokkema and Greiff, 2017), the EFA and CFA were each performed on half of the participant sample, which was divided into two groups of 863 people.

Based on the information from Cattell’s scree test, five items resulted in the factor loadings structure matrix, which is shown in Table 2 and depicts the model matrix with saturations on the factor and Uniqueness. The factorial loadings were all statistically significant and varied from 0.554 to 0.780. (*p* 0.001). The CRI (0.727) was also acceptable.

**TABLE 2:** Factor loadings structure matrix.

	Factor loadings	Uniqueness
DRS 1	0.780	0.392
DRS 2	0.664	0.559
DRS 3	0.750	0.437
DRS 4	0.554	0.693
DRS 5	0.761	0.421

Extraction Method: Maximum Likelihood. Cumulative variance: 60.2%.

**FIGURE 1:**
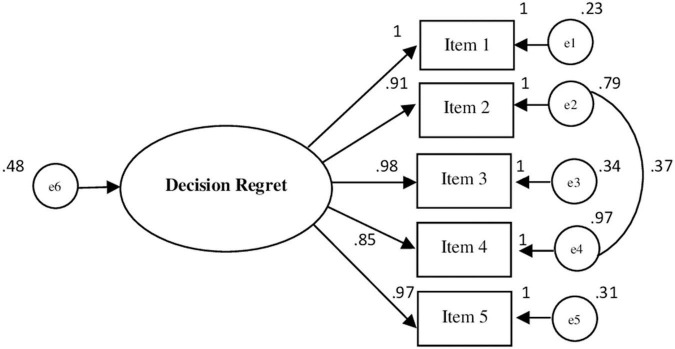
Path diagram of the confirmatory factor analysis (5 items). Chi-square = 6.019; χ^2^df = 1.506; CFI = 0.999; TLI = 0.997; RMSEA = 0.024 and RMSEA 90% CI [0.00–0.051].

The CFA (see Figure 1) revealed that a model with one factor and five items, specifying the error covariance between item 2 and item 4, provided overall good indices of data adaptation: χ^2^ = 6.019; p = 0.198; χ^2^/df = 1.506; GFI = 0.997; AGFI = 0.959; CFI = 0.999; TLI = 0.997; RMSEA = 0.024; and RMSEA 90% CI [0.000–0.051]; p-close = 0.855; NFI = 0.996.

Item statistics and internal consistency are shown in Table 3. Ceiling effects were present, ranging from 26.3 percent (item 4) to 47.4 percent (item 1). Item 1 was the “most regrettable,” with a mean score of 4.28, while item 4 was the “least regrettable,” with a mean score of 3.53. The overall mean score was 73.31 (0–100), with a standard deviation of 19.01. The scale exhibited satisfactory internal consistency with α = 0.81 and ω = 0.81.

**TABLE 3:** DRS item statistics and reliability.

		Item 1	Item 2	Item 3	Item 4	Item 5	Overall
Response	Strongly agree (floor effect)	0	3.7	0	5.2	0	
	Agree	2.5	14.0	4.6	17.2	3.7	
	Neither agree nor disagree	14.1	23.6	22.4	23.2	22.7	
	Disagree	36.0	28.9	33.5	28.1	33.8	
	Strongly disagree (ceiling effect)	47.4	29.8	39.5	26.3	39.9	
Item statistics	Mean	4.28	3.67	4.08	3.53	4.10	73.31
	Standard deviation	0.80	1.15	0.89	1.20	0.87	19.01
	Skewness	–0.851	–0.476	–0.545	–0.370	–0.524	–0.154
	Kurtosis	–0.040	–0.726	–0.717	–0.882	–0.753	–0.995
Internal reliability	Alpha if item drop	0.78	0.77	0.78	0.81	0.78	
	Item-total correlation	0.65	0.63	0.63	0.65	0.54	
	Cronbach’s alpha						0.81
	95% IC						[0.797, 0.835]
	McDonald’s omega						0.81
	95% IC						[0.791, 0.831]
	Gutmann’s lamda						0.81
	95% IC						[0.795, 0.833]
	Average inter-item correlation						0.496
	95% IC						[0.463, 0.529]

The scores of items 2 and 4 have been reversed. Strongly agree means low regret and strongly disagree means high regret. DRS: Item 1, It was the right decision; Item 2, I regret the choice that was made; Item 3, I would go for the same choice if I had to do it over again; Item 4, The choice did me a lot of harm; and Item 5, The decision was a wise one.

The measurement invariance of the DRS factorial structure was also tested by gender. We examined four nested models with varying degrees of constraint. The goodness-of-fit indices of the multidimensional model by gender and layered models of invariance are shown in Table 4 in ascending order of restriction level. The DRS was shown to have high gender invariance, with an outstanding one-dimensional model fit for male and female.

**TABLE 4:** Tested models and goodness-of-fit indices.

	χ^2^	*df*	Δ χ^2^	Δ *df*	CFI	TLI	RMSEA	Δ CFI	Δ TLI	Δ RMSEA
**Models in each group**										
Gender										
Male	2.901	4			1.00	1.00	0.000			
Female	7.131	4			0.996	0.990	0.042			
Gender										
Configural	10.032*	8	–	–	0.999	0.997	0.024	–	–	–
Metric	13.959*	12	3.927	4	0.999	0.998	0.019	0.000	0.001	–0.005
Scalar	20.980*	16	7.021	4	0.997	0.996	0.027	–0.002	–0.002	0.008
Strict	32.074*	22	11.094	6	0.994	0.994	0.033	–0.002	–0.002	0.006

*df*, degrees of freedom; χ^2^, Chi square; Δχ^2^, difference in Chi square; Δ*df*, difference in degrees of freedom; CFI, comparative fit index; TLI, Tucker-Lewis index; RMSEA, root mean square error of approximation; ΔCFI, difference in comparative fit index; ΔTLI, difference in Tucker-Lewis index; ΔRMSEA, difference in root mean square error of approximation *p < 0.001.

These data imply that the latent means may be compared by gender. Females’ latent mean values were set to zero, and females exhibited greater latent mean values of Regret in this study, as can be seen in Table 5.

**TABLE 5:** Gender latent mean values.

Variable	Factor	Mean	*SE*	CR	*P*
Gender (male)*	Regret	–0.99	0.08	–12.33	<0.001

SE, standard error; CR, critical ratio. *Reference variable is female.

To assess convergent validity, correlation coefficients with the GDMS (Scott and Bruce, 1995) and the Adjustment Mode Scale (Higgins et al., 2003; Pierro et al., 2006) were examined. A new sample of 808 people was used to assess convergent validity: 444 men (55%) and 364 women (45%) with a mean age of 23.54 years and *SD* = 4.05. For the predicted directions of these correlations, two hypotheses were proposed: (1) the higher the DRS score, the higher the Rational and Intuitive choice styles and the lower the Avoidant, Dependent and Spontaneous decision-making styles; (2) the higher the RDS score, the higher the Locomotion and the lower the Evaluation regulation mode.

As shown in Table 6, the results for the first hypothesis confirmed the assumed directions of correlation for the Rational, Avoidant, Intuitive, and Spontaneous decision styles; while for the second hypothesis, the expected association with the Locomotion Mode was found, as well as an indirect direction with the Assessment Mode. For these convergent administrations, the DRS McDonald’s and Alpha coefficients were 0.82 (95% IC 0.808, 0.835) and 0.82 (95% IC 0.804, 0.831), respectively.

**TABLE 6:** Pearson’s correlations.

		Rational	Avoidant	Dependent	Intuitive	Spontaneous	Locomotion
Regret (DRS)	1						
Rational (GDMS)	0.329**	1					
Avoidant (GDMS)	–0.314**	–0.143**	1				
Dependent (GDMS)	–0.041	0.203**	0.345**	1			
Intuitive (GDMS)	0.101**	0.183**	0.117**	0.162**	1		
Spontaneous (GDMS)	–0.142**	–0.262**	0.121**	–0.111**	0.347**	1	
Locomotion (SRM)	0.335**	0.505**	–0.320**	0.007	0.245**	–0.014	1
Assessment (SRM)	–0.074*	0.269**	–0.283**	0.234**	0.125**	0.026	0.297**

**Correlation is significant at the 0.01 level (2-tailed). *Correlation is significant at the 0.05 level (2-tailed).

## Discussion

The DRS was used to assess the psychometric features of decision regret in ordinary life settings. Although prior research has mostly used the scale with patients in the context of medical decision-making, our contribution shows that the instrument may be used in non-medical situations and yet preserve strong metric features. The CFA showed overall good indices of adaptation to data, although specifying the covariance of error between item 2 and item 4. Chinese (Xu et al., 2020) and German (Haun et al., 2019) validations also achieved a similar model with the error covariance on the same items. Moreover, the scale revealed good internal consistency and strong gender invariance among the sample of university students used, while the convergence analysis reported significant correlations with the instruments that measure the person’s decision-making style and regulatory modes.

At present the only other instrument available for the measurement of regret on the Italian psychometric scene is that of Marcatto and Ferrante (2008), which however, focuses mainly on the person’s emotional reaction, expressly distinguishing between regret and disappointment, and on the identification of the causal attribution orientation (internal/external). However, critical issues remain in the discrimination of the cognitive antecedents of the emotional responses of individuals placed in specific contexts. Therefore, considering the persisting difficulties linked to the fact that the construct presents abstract and complex aspects, the RDS instead has the advantage of focusing on a functional measure of regret (DRS) that evaluates both the aspect of the option and the outcome of the choice, deliberately leaving aside the complex discrimination of the person’s emotional response.

According to the scale’s authors (Brehaut et al., 2003), people who make decisions under less-than-ideal circumstances, such as inadequate knowledge or hasty or insufficient decision-making methods, are more likely to regret their decisions if bad results occur. They also hypothesized that the degree of decisional conflict present at the time of the decision could have an impact on regret. Decisional conflict is characterized as the incapacity to select between numerous options due to the risks associated with different outcomes, a lack of assistance in the decision-making process, or the need to make value judgments about potential gains or losses. In the framework of our study, the decisional conflict was rather traced back to the tension that can be generated by choices between alternatives pursuing different goals in the given situation (personal gain, personal pleasure and satisfaction, moral obligation, social expectation). A preliminary presentation of this approach was illustrated in a previous study by Diotaiuti et al. (2020a).

The present results showed that the DRS scale is able to measure the regret generated by the person’s immersion in a problematic scenario of everyday life that requires a choice between alternatives characterized by different goals.

In the face of an extensive literature that has highlighted a significant difference in the frequency and quality of the experience of regret between genders (Roese et al., 2009; Coats et al., 2012; Newton et al., 2012; Galperin et al., 2013), the test of invariance in measurement with DRS reassures the reliability of assessing regret in both genders. The gender study of measurement invariance showed crucial features connected to probable gender disparities in a person’s Regret experience. When the values of the latent averages in the component forming the DRS instrument were examined, it was discovered that women reported values suggesting a larger feeling of regret in settings of everyday decision than males. In the literature, for example, Roese et al. (2006) illustrate some basic motivational differences in gender regret: women focus more on thinking about actions that should have been avoided, whereas men reflect more frequently on actions that should have been taken to produce a better situation. Regardless of the motivational orientation, which may be different, DRS is a reliable measure of choice regret in the various problematic situations of everyday life in both men and women.

Convergence analysis showed that participants with higher regret scores were those who favored a rational decision-making style, while lower regret scores correlated with avoidant and spontaneous styles. In the literature the link between regret and rationality in choices is referred on the one hand to the assumption of full responsibility by the person who prepares the choice through a meticulous evaluation and comparison of the relevant information in the situation. The decision-maker reaches the conclusion of the decision-making process with difficulty and, faced with a possible suboptimal outcome (real or anticipated), is prompted to regretfully reconsider the quality of the choice made and to reproach himself for not being meticulous enough (Bourgeois-Gironde, 2010; Jurasova and Spajdel, 2011; Bourgeois-Gironde, 2017). On the other hand, the literature points to the presence of situations in which there is necessarily a degree of uncertainty about the components that characterize them and the decision-making calculation is structurally incomplete and can only be a mere probable estimate of the outcomes; the final uncertainty would however, drive the rational decision-maker who tries to refine the decision-making process by adaptively using the knowledge of negative experiences (Bell, 1982; Reb, 2008; Joseph-Williams et al., 2011).

Other significant studies have also emphasized the influence of regret on the decision-making process (Abraham and Sheeran, 2004; Pieters and Zeelenberg, 2007; O’Connor et al., 2014, 2015; Speck et al., 2016). However, it should also be considered, especially in everyday contexts, that there are situations in which a direct comparison between the choice alternatives is not possible, since the cognitive and affective antecedents that support the plausibility of the choice of the single alternative are different (Kujawski, 2005). In many cases the alternatives might still have adequate and relevant rational justifications, so that an absolute criterion of discrimination would be lacking. In such situations of rational “equivalence” the choice is made more difficult by the fact that there are no overriding reasons, but the decision-maker feels that each alternative can find its own justification in that context. Therefore he/she faces the choice with an explicit experience of regret, with the awareness that the choice made may not be final (Connolly and Zeelenberg, 2002). Therefore, it could be said that the rational decision-maker, when faced with uncertain and complex situations, does not shy away from regret, but rather, with his or her style of decision-making thinking, activates the experience of regret and preserves it by keeping the evaluation process open to the entire context and in a broader temporal perspective.

Regret experiences stimulate more thoughtful and thorough decision making and the formation of future behavioral goals (Tsiros and Mittal, 2000; Reb, 2008; Diotaiuti et al., 2021). After experiencing regret, people are more likely to recognize and correct previous mistakes (Zeelenberg and Pieters, 1999; Mallia et al., 2020). In extreme decision-making contexts, the phenomenon of experts’ Decision Inertia has also been highlighted; these get stuck in the evaluation cycle of alternatives that nevertheless imply important and unavoidable negative consequences (Power and Alison, 2018).

In the convergence analysis of our study, a negative direction emerged between regret and avoidant decision-making style. As indicated by Anderson (2003), Decision avoidance is an approach to choice in which people avoid making a decision, postpone a conclusion, or make a decision that does not require action or change. The result of avoidance is a negative emotional state that tends to be lower than that initially triggered by the decision problem. Although avoidance allows the negative emotional weight of the consequences of the choice to be contained, studies have shown a recurrent association with erroneous decision-making strategies in the medium and long term (Bruine de Bruin et al., 2007).

The avoidant style is also linked to a low capacity for self-regulation, low self-esteem, and difficulties in taking initiatives (Thunholm, 2004). Hunt et al. (2009) highlighted the connection between the propensity to regret and the aversive and avoidant aspects of indecisiveness in individuals: while the former is an aversion to making decisions, which emerges as threat-oriented cognition and negative emotion when making judgments, the latter is a generalized motivation to avoid making decisions and having difficulty doing so. In our study the avoidant approaches the choice with a lower level of responsibility and therefore the eventual experience of regret is equally contained (see also Ordóñez and Connolly, 2000; Zeelenberg et al., 2000).

A negative association with the experience of regret has also emerged in the case of the spontaneous decision-making style. The spontaneous style is defined by a sense of urgency and a desire to complete the decision-making process as rapidly as possible based on the available options. There is no explicit attention to the evaluation of the effects at the time of the decision, therefore anticipatory regret is not present. However, the literature has shown an association with the lower decision-making competence of the avoidant and spontaneous styles (Bavol’ár and Orosová, 2015).

With regard to regulatory modes, the relationship between regret and locomotion was positive. Since locomotion indicates a pronounced orientation to the future and to the goal to be achieved, the decision-maker would like to maximize the result when making a choice, and the subsequent regret indicates dissatisfaction, a continuous search for improvement in the results and a heartfelt and prolonged involvement with the goal to be achieved. However, this result is contrary to previous findings in the literature in which the direction of the association between locomotion and regret was instead inverse (Pierro et al., 2008; Panno et al., 2015; Kruglanski et al., 2018).

The data also showed a significant correlation between the rational decision-making style and locomotion. The avoidant style registered a positive direction with the regulatory mode of assessment, thus the decision maker’s increased dependence on external pressures and sensitivity to criticism, and whose fear could probably fuel avoidant or impulsive modes of choice in order to attempt to contain the discomfort of post-decision regret. Overall, the directions of association that emerged with the instruments measuring decision-making style and regulatory modes point to an interesting predictive measure of a person’s decision-making and self-regulatory orientation through the assessment of regret using the DRS.

## Limitations of the study

This contribution should also be considered in the light of a few limitations. Firstly, the use of a convenience sample consisting of university students should be supplemented with participants from other age groups. A further test of the validity of the scale could include the evaluation following real, concrete choice situations rather than the use of identification scenarios. Since the metric validity of the scale was tested here with general decision-making scenarios and everyday student life situations, further confirmation of the assessment properties of regret should be acquired by extending it to specific choice domains, such as professional choice, university choice, choice in the sentimental sphere (marriage, separation, divorce), choice of the purchase of goods and services, as well as clinical choice, which was the object of study in the original construction of the scale. The convergence analysis could perhaps also have included the comparison with another instrument for the evaluation of regret, even though at the time the study was carried out only Marcatto and Ferrante (2008) was present in the Italian context, which however, is more oriented toward the distinction between regret and disappointment and the orientation of causal attribution. Future studies could also better explore the relationship between individual decisional regret and shared counterfactual narratives within different communicative contexts (Sunwolf, 2006; Diotaiuti et al., 2020b; Kim, 2020).

## Conclusion

The present study proposes the use of the DRS for the general measurement of regret in non-clinical contexts. The excellent psychometric properties found foreshadow a reliable use in various contexts where knowledge of post-decisional attitude becomes important: school, university and professional orientation, marketing studies, relational choices, as well as for use in the field of research.

## Data availability statement

The raw data supporting the conclusions of this article will be made available by the authors, without undue reservation.

## Ethics statement

The studies involving human participants were reviewed and approved by the Institutional Review Board (IRB) of the University of Cassino and Southern Lazio. The participants provided their written informed consent to participate in this study.

## Author contributions

PD, GV, and SM designed the study, analyzed the data, and discussed the results. PD, GV, and AG drafted the manuscript. AC and FL revised the manuscript. All authors contributed to the article and approved the submitted version.

## References

[B1] Abraham C. Sheeran P. (2004). Deciding to exercise: The role of anticipated regret. *Br. J. Health Psychol.* 9 269–278. 10.1348/135910704773891096 15125809

[B2] Advani P. G. Lei X. Swanick C. W. Xu Y. Shen Y. Goodwin N. A. (2019). Local therapy decisional regret in older women with breast cancer: A population-based study. *Int. J. Radiat. Oncol. Biol. Phys.* 104 383–391. 10.1016/j.ijrobp.2019.01.089 30716524 PMC6624842

[B3] Anderson C. J. (2003). The psychology of doing nothing: Forms of decision avoidance result from reason and emotion. *Psychol. Bull.* 129 139–167. 10.1037/0033-2909.129.1.139 12555797

[B4] Barbaranelli C. Ingoglia S. (eds) (2013). *I Modelli di Equazioni Strutturali: Temi e Prospettive [Structural Equation Models: Issues and Perspectives].* Milano: LED.

[B5] Bavol’ár J. Orosová O. G. (2015). Decision-making styles and their associations with decision-making competencies and mental health. *Judgm. Decis. Mak.* 10 115–122.

[B6] Beaton D. E. Bombardier C. Guillemin F. Ferraz M. B. (2000). Guidelines for the process of cross-cultural adaptation of self-report measures. *Spine* 25 3186–3191. 10.1097/00007632-200012150-00014 11124735

[B7] Becerra Pérez M. M. Menear M. Brehaut J. C. Légaré F. (2016). Extent and Predictors of Decision Regret about Health Care Decisions: A Systematic Review. *Med. Decis. Making* 36 777–790. 10.1177/0272989X16636113 26975351

[B8] Bell D. E. (1982). Regret in decision making under uncertainty. *Oper. Res.* 30 961–981. 10.1287/opre.30.5.961 19642375

[B9] Bonaccio S. Girard A. J. (2015). Measuring decision-making regret among French populations: Translation and validation of the Regret Scale. *Eur. J. Psychol. Assess.* 31:143. 10.1027/1015-5759/a000219

[B10] Bourgeois-Gironde S. (2010). Regret and the rationality of choices. *Philos. Trans. R. Soc. Lond. B Biol. Sci.* 365 249–257. 10.1098/rstb.2009.0163 20026463 PMC2827451

[B11] Bourgeois-Gironde S. (2017). “How regret moves individual and collective choices towards rationality,” in *Handbook Of Behavioural Economics And Smart Decision-Making*, ed. Altman M. (Cheltenham: Edward Elgar Publishing), 188–204. 10.4337/9781782549598

[B12] Brehaut J. C. O’Connor A. M. Wood T. J. Hack T. F. Siminoff L. Gordon E. (2003). Validation of a decision regret scale. *Med. Decis. Making* 23 281–292. 10.1177/0272989X03256005 12926578

[B13] Bruine de Bruin W. Parker A. M. Fischhoff B. (2007). Individual differences in adult decision-making competence. *J. Pers. Soc. Psychol.* 92 938–956. 10.1037/0022-3514.92.5.938 17484614

[B14] Buchanan J. Summerville A. Lehmann J. Reb J. (2016). The Regret Elements Scale: Distinguishing the affective and cognitive components of regret. *Judgm. Decis. Mak.* 11 275–286. 10.1159/000178755 19052451

[B15] Byrne B. M. (2001). *Structural Equation Modeling with AMOS: Basic Concepts, Applications, and Programming.* Mahwah, NJ: Lawrence Erlbaum Associates.

[B16] Byrne R. M. J. McEleney A. (2000). Counterfactual thinking about actions and failures to act. *J. Exp. Psychol.* 26 1318–1331. 10.1037/0278-7393.26.5.1318 11009260

[B17] Calderon C. Ferrando P. J. Lorenzo-Seva U. Higuera O. Ramon Y. Cajal T. (2019). Validity and Reliability of the Decision Regret Scale in Cancer Patients Receiving Adjuvant Chemotherapy. *J. Pain Symptom Manag.* 57 828–834. 10.1016/j.jpainsymman.2018.11.017 30639730

[B18] Cheung G. W. Rensvold R. B. (2002). Evaluating goodness-of-fit indexes for testing measurement invariance. *Struct. Equ. Model.* 9 233–255. 10.1207/S15328007SEM0902_5 33486653

[B19] CoatsS.HarringtonJ. T.BeaubouefM.LockeH. (2012). Sex differences in relationship regret: The role of perceived mate characteristics. *Evol. Psychol.* 10 422–442. 10.1177/14747049120100030422947670

[B20] Connolly T. Zeelenberg M. (2002). Regret in decision making. *Curr. Dir. Psychol. Sci.* 11 212–216. 10.1111/1467-8721.00203

[B21] Coricelli G. Critchley H. D. Joffily M. O’Doherty J. P. Sirigu A. Dolan R. J. (2005). Regret and its avoidance: A neuroimaging study of choice behavior. *Nat. Neurosci.* 8 1255–1262. 10.1038/nn1514 16116457

[B22] Creyer E. H. Ross W. T. (1999). The Development and Use of a Regret Experience Measure to Examine the Effects of Outcome Feedback on Regret and Subsequent Choice. *Mark. Lett.* 10 373–386. 10.1023/A:1008166305455

[B23] DeVellis R. F. (2017). *Scale Development: Theory and Applications*, 4th Edn. Thousand Oaks, CA: Sage.

[B24] Di Fabio A. (2007). General decision making style (GDMS): Un primo contributo alla validazione italiana. [General Decision Making Style (GDMS): A first contribution to Italian validation]. *GIPO, Giornale Italiano di Psicologia dell’Orientamento* 8, 17–25.

[B25] Diotaiuti P. Valente G. Mancone S. (2021). Validation study of the italian version of temporal focus scale: Psychometric properties and convergent validity. *BMC Psychol.* 9, 19. 10.1186/s40359-020-00510-5 33522963 PMC7851924

[B26] Diotaiuti P. Valente G. Mancone S. Grambone A. (2020a). Resist or Give in to an Alternative: Post-Decisional Evaluations of Cost, Value and Regret in the Choice. *Psychology* 11:245. 10.4236/psych.2020.112016

[B27] Diotaiuti P. Valente G. Mancone S. Grambone A. (2020b). Psychometric properties and a preliminary validation study of the Italian brief version of the communication styles inventory (CSI-B/I). Front. Psychol. 11, 1421. 10.3389/fpsyg.2020.01421 32655460 PMC7323508

[B28] Fokkema M. Greiff S. (2017). How Performing PCA and CFA on the Same Data Equals Trouble Overfitting in the Assessment of Internal Structure and Some Editorial Thoughts on It. *Eur. J. Psychol. Assess.* 33 399–402. 10.1027/1015-5759/a000460

[B29] Gaafar M. E. S. Abd El Salam R. M. El Kholy S. E. H. (2018). Relationship between Decision Making Styles and Life Regrets among Community Dwelling Older Adults. *Alex. Sci. Nurs. J.* 20 1–12. 10.1093/schbul/sbq167 21248277 PMC3406533

[B30] Galperin A. Haselton M. G. Frederick D. A. Poore J. von Hippel W. Buss D. M. (2013). Sexual regret: Evidence for evolved sex differences. *Arch. Sex. Behav.* 42 1145–1161. 10.1007/s10508-012-0019-3 23179233

[B31] Gilovich T. Medvec V. H. (1994). The temporal pattern to the experience of regret. *J. Pers. Soc. Psychol.* 67 357–365. 10.1037/0022-3514.67.3.357 7965599

[B32] Godin G. Sheeran P. Conner M. Germain M. Blondeau D. Gagné C. (2005). Factors explaining the intention to give blood among the general population. *Vox Sang.* 89 140–149. 10.1111/j.1423-0410.2005.00674.x 16146506

[B33] Goel V. Sawka C. A. Thiel E. C. Gort E. H. O’Connor A. M. (2001). Randomized trial of a patient decision aid for choice of surgical treatment for breast cancer. *Med. Decis. Making* 21 1–6. 10.1177/0272989X0102100101 11206942

[B34] Haun M. W. Schakowski A. Preibsch A. Friederich H. C. Hartmann M. (2019). Assessing decision regret in caregivers of deceased German people with cancer—A psychometric validation of the Decision Regret Scale for Caregivers. *Health Expect.* 22 1089–1099. 10.1111/hex.12941 31368210 PMC6803409

[B35] Hetts J. J. Boninger D. S. Armor D. A. Gleicher F. Nathanson A. (2000). The influence of anticipated counterfactual regret on behavior. *Psychol. Mark.* 17 345–368. 10.1002/(SICI)1520-6793(200004)17:4<345::AID-MAR5<3.0.CO;2-M

[B36] Higgins E. T. Kruglanski A. W. Pierro A. (2003). Regulatory mode: Locomotion and assessment as distinct orientations. *Adv. Exp. Soc. Psychol.* 35 293–344. 10.1016/S0065-2601(03)01005-0

[B37] Hoelzl E. Loewenstein G. (2005). Wearing out your shoes to prevent someone else from stepping into them: Anticipated regret and social takeover in sequential decisions. *Organ. Behav. Hum. Decis. Process.* 98 15–27. 10.1016/j.obhdp.2005.04.004

[B38] Hu L. T. Bentler P. M. (1999). Cut-off criteria for fit indexes in covariance structure analysis: Conventional criteria versus new alternatives. *Struct. Equ. Model.* 6 1–55. 10.1080/10705519909540118

[B39] Hunt K. France E. Ziebland S. Field K. Wyke S. (2009). ‘My brain couldn’t move from planning a birth to planning a funeral’: A qualitative study of parents’ experiences of decisions after ending a pregnancy for fetal abnormality. *Int. J. Nurs. Stud.* 46, 1111–1121. 10.1016/j.ijnurstu.2008.12.004 19147141

[B40] Jonassen D. H. (2012). Designing for decision making. *Educ. Technol. Res. Dev.* 60 341–359. 10.1007/s11423-011-9230-5

[B41] Joseph-Williams N. Edwards A. Elwyn G. (2011). The importance and complexity of regret in the measurement of ‘good’ decisions: A systematic review and a content analysis of existing assessment instruments. *Health Expect.* 14 59–83. 10.1111/j.1369-7625.2010.00621.x 20860776 PMC5060557

[B42] Jurasova K. Spajdel M. (2011). The role of regret in rational decision making. *Stud. Psychol.* 53 169–174.

[B43] Kahneman D. Miller D. (1986). Norm theory: Comparing reality to its alternative. *Psychol. Rev.* 93 136–153. 10.1037/0033-295X.93.2.136

[B44] Keaveney S. M. Huber F. Herrmann A. (2007). A model of buyer regret: Selected prepurchase and postpurchase antecedents with consequences for the brand and the channel. *J. Bus. Res.* 60 1207–1215. 10.1016/j.jbusres.2006.07.005

[B45] Kim J. (2020). The impact of narrative strategy on promoting HPV vaccination among college students in Korea: The role of anticipated regret. *Vaccines* 8, 176. 10.3390/vaccines8020176 32290099 PMC7349232

[B46] Kruglanski A. W. Chernikova M. Jasko K. (2018). “The forward rush: On locomotors’ future focus,” in *The Psychology Of Thinking About The Future*, eds Oettingen G. Sevincer A. T. Gollwitzer P. (New York, NY: The Guilford Press), 405–422.

[B47] Kruglanski A. W. Thompson E. P. Higgins E. T. Atash M. N. Pierro A. Shah J. Y. (2000). ‘To “do the right thing” or to “just do it”: Locomotion and assessment as distinct self-regulatory imperatives’. *J. Pers. Soc. Psychol.* 79 793–815. 10.1037//0022-3514.79.5.793 11079242

[B48] Kujawski E. (2005). A reference-dependent regret model for deterministic tradeoff studies. *Syst. Eng.* 8 119–137. 10.1002/sys.20027

[B49] Lauriola M. Levin I. P. (2001). Personality traits and risky decision-making in a controlled experimental task: An exploratory study. *Pers. Individ. Differ.* 31 215–226. 10.1016/S0191-8869(00)00130-6

[B50] Liu J. Hunter S. Zhu J. Lee R. L. T. Chan S. W. C. (2022). Decision regret regarding treatments among women with early-stage breast cancer: A systematic review protocol. *BMJ Open* 12:e058425. 10.1136/bmjopen-2021-058425 35301213 PMC8932263

[B51] Mallia L. Chirico A. Zelli A. Galli F. Palombi T. Bortoli L. (2020). The implementation and evaluation of a media literacy intervention about PAES use in sport science students. *Front. Psychol.* 11, 368. 10.3389/fpsyg.2020.00368 32265771 PMC7105711

[B52] Marcatto F. Ferrante D. (2008). The Regret and Disappointment Scale: An instrument for assessing regret and disappointment in decision making. *Judgm. Decis. Mak.* 3 87–99.

[B53] Mazzocco K. (2008). “Emozioni e decisione,” in *Psicologia del giudizio e della decisione [Psychology of judgement and decision-making]*, eds Bonini N. Del Missier F. Rumiati R. (Bologna: Il Mulino), 151–170.

[B54] McConnell A. R. Niedermeier K. E. Leibold J. M. El-Alayli A. G. Chin P. P. Kuiper N. M. (2000). What if I find it cheaper someplace else?: Role of prefactual thinking and anticipated regret in consumer behavior. *Psychol. Mark.* 17 281–298. 10.1002/(SICI)1520-6793(200004)17:4<281::AID-MAR2<3.0.CO;2-5

[B55] Miller D. T. Taylor B. R. (1995). “Counterfactual thought, regret, and superstition: How to avoid kicking yourself,” in *What Might Have Been*, eds Roese N. J. Olson J. M. (London: Psychology Press), 317–344.

[B56] Mundfrom D. J. Shaw D. G. Ke T. L. (2005). Minimum sample size recommendations for conducting factor analyses. *Int. J. Test.* 5 159–168. 10.1207/s15327574ijt0502_4

[B57] Newton N. Torges C. Stewart A. (2012). Women’s regrets about their lives: Cohort differences in correlates and contents. *Sex Roles* 66 530–543. 10.1007/s11199-012-0126-6

[B58] O’Connor E. McCormack T. Beck S. R. Feeney A. (2015). Regret and adaptive decision making in young children. *J. Exp. Child Psychol.* 135 86–92. 10.1016/j.jecp.2015.03.003 25843700

[B59] O’Connor E. McCormack T. Feeney A. (2014). Do children who experience regret make better decisions? A developmental study of the behavioral consequences of regret. *Child Dev.* 85 1995–2010. 10.1111/cdev.12253 24773388 PMC4282021

[B60] Ordóñez L. D. Connolly T. (2000). Regret and responsibility: A reply to Zeelenberg et al. (1998). *Organ. Behav. Hum. Decis. Process.* 81 132–142. 10.1006/obhd.1999.2834 10631072

[B61] Panno A. Lauriola M. Pierro A. (2015). Regulatory mode and risk-taking: The mediating role of anticipated regret. *PLoS One* 10:e0143147. 10.1371/journal.pone.0143147 26580960 PMC4651368

[B62] Papé L. Martinez L. F. (2017). Past and future regret and missed opportunities: An experimental approach on separate evaluation and different time frames. *Psicologia* 30:20. 10.1186/s41155-017-0074-8 32026096 PMC6974344

[B63] Pierro A. Kruglanski A. W. Higgins E. T. (2006). Regulatory mode and the joys of doing: Effects of ‘locomotion’and ‘assessment’on intrinsic and extrinsic task-motivation. *Eur. J. Pers.* 20 355–375. 10.1002/per.600

[B64] Pierro A. Leder S. Mannetti L. Higgins E. T. Kruglanski A. W. Aiello A. (2008). Regulatory mode effects on counterfactual thinking and regret. *J. Exp. Soc. Psychol.* 44 321–329. 10.1016/j.jesp.2007.06.002

[B65] Pieters R. Zeelenberg M. (2007). A theory of regret regulation 1.1. *J. Consum. Psychol.* 17 29–35. 10.1207/s15327663jcp1701_6 26627889

[B66] Power N. Alison L. (2018). Decision inertia in critical incidents. *Eur. Psychol.* 24 209–218. 10.1027/1016-9040/a000320

[B67] Raykov T. (1997). Estimation of composite reliability for congeneric measures. *Appl. Psychol. Meas.* 21 173–184. 10.1177/01466216970212006

[B68] Reb J. (2008). Regret aversion and decision process quality: Effects of regret salience on decision process carefulness. *Organ. Behav. Hum. Decis. Process.* 105 169–182. 10.1016/j.obhdp.2007.08.006

[B69] Roese N. J. Epstude K. A. I. Fessel F. Morrison M. Smallman R. Summerville A. (2009). Repetitive regret, depression, and anxiety: Findings from a nationally representative survey. *J. Soc. Clin. Psychol.* 28 671–688. 10.1521/jscp.2009.28.6.671

[B70] Roese N. J. Pennington G. L. Coleman J. Janicki M. Li N. P. Kenrick D. T. (2006). Sex differences in regret: All for love or some for lust? *Pers. Soc. Psychol. Bull.* 32 770–780. 10.1177/0146167206286709 16648202 PMC2293329

[B71] Schermelleh-Engel K. Moosbrugger H. Müller H. (2003). Evaluating the fit of structural equation models: Tests of significance and descriptive goodness-of-fit measures. *Methods Psychol. Res. Online* 8 23–74.

[B72] Schwartz B. Ward A. Monterosso J. Lyubomirsky S. White K. Lehman D. R. (2002). Maximizing versus satisficing: Happiness is a matter of choice. *J. Pers. Soc. Psychol.* 83:1178. 10.1037/0022-3514.83.5.1178 12416921

[B73] Scott S. G. Bruce R. A. (1995). Decision-making style: The development and assessment of a new measure. *Educ. Psychol. Meas.* 55 818–831. 10.1177/0013164495055005017

[B74] Seta C. E. Seta J. J. McElroy G. T. Hatz J. (2008). Regret: The roles of consistency-fit and counterfactual salience. *Soc. Cogn.* 26 700–719. 10.1521/soco.2008.26.6.700

[B75] Sheeran P. Orbell S. (1999). Augmenting the theory of planned behavior: Roles for anticipated regret and descriptive norms. *J. Appl. Soc. Psychol.* 29 2107–2142. 10.1111/j.1559-1816.1999.tb02298.x

[B76] Speck R. M. Neuman M. D. Resnick K. S. Mellers B. A. Fleisher L. A. (2016). Anticipated regret in shared decision-making: A randomized experimental study. *Perioper. Med.* 5:5. 10.1186/s13741-016-0031-6 26941952 PMC4776353

[B77] Sugden R. (1985). Regret, recrimination and rationality. *Theory Decis.* 19 77–99. 10.1007/BF00134355

[B78] Sunwolf (2006). Decisional regret theory: Reducing the anxiety about uncertain outcomes during group decision making through shared counterfactual storytelling. *Commun. Stud.* 57, 107–134. 10.1080/10510970600666750

[B79] Tanno K. Bito S. Isobe Y. Takagi Y. (2016). Validation of a Japanese version of the decision regret scale. *J. Nurs. Meas.* 24:E44-54. 10.1891/1061-3749.24.1.44 27103244

[B80] Telatar T. G. Ozel C. S. Turgut A. Kınlı Ö (2021). Turkish version methodological validation study of the Decision Regret Scale. *Ethiop. J. Health Dev.* 35. Available online at: https://ejhd.org/index.php/ejhd/article/view/4820

[B81] Thunholm P. (2004). Decision-making style: Habit, style or both? *Pers. Individ. Differ.* 36 931–944. 10.1016/S0191-8869(03)00162-4

[B82] Tsiros M. (1998). Effect of regret on post-choice valuation: The case of more than two alternatives. *Organ. Behav. Hum. Decis. Process.* 76 48–69. 10.1006/obhd.1998.2793 9756739

[B83] Tsiros M. Mittal V. (2000). Regret: A model of its antecedents and consequences in consumer decision making. *J. Consum. Res.* 26, 401–417. 10.1086/209571

[B84] Ueichi H. Kusumi T. (2004). Change in feelings of regret over time: Relation to decision-making style, behavior, and coping methods. *Jpn. J. Psychol.* 74 487–495. 10.4992/jjpsy.74.487 15112503

[B85] Wilding S. Downing A. Selby P. Cross W. Wright P. Watson E. K. (2020). Decision regret in men living with and beyond nonmetastatic prostate cancer in the United Kingdom: A population-based patient-reported outcome study. *Psycho Oncol.* 29 886–893. 10.1002/pon.5362 32065691 PMC7317932

[B86] Wong K. F. E. Kwong J. Y. Y. (2007). The role of anticipated regret in escalation of commitment. *J. Appl. Psychol.* 92 545–554. 17371099 10.1037/0021-9010.92.2.545

[B87] Xu R. H. Zhou L. M. Wong E. L. Wang D. Chang J. H. (2020). Psychometric Evaluation of the Chinese Version of the Decision Regret Scale. *Front. Psychol.* 11:583574. 10.3389/fpsyg.2020.583574 33424697 PMC7793926

[B88] Zeelenberg M. Pieters R. (1999). Comparing service delivery to what might have been: Behavioral responses to regret and disappointment. *J. Serv. Res.* 2 86–97. 10.1177/109467059921007

[B89] Pieters M. Zeelenberg R. (2007). A theory of regret regulation 1.0. *J. Consum. Psychol.* 17 3–18. 10.1207/s15327663jcp1701_3 PMC2435165 18568095

[B90] Zeelenberg M. van Dijk W. W. Manstead A. S. R. (1998). Reconsidering the relation between regret and responsibility. *Organ. Behav. Hum. Decis. Process.* 74 254–272. 10.1006/obhd.1998.2780 9719654

[B91] Zeelenberg M. van Dijk W. W. Manstead A. S. R. van der Plight J. (2000). On bad decisions and disconfirmed expectancies: The psychology of regret and disappointment. *Cogn. Emot.* 14 521–541. 10.1080/026999300402781

